# Unraveling Natural Products’ Role in Osteoarthritis Management—An Overview

**DOI:** 10.3390/antiox9040348

**Published:** 2020-04-23

**Authors:** Georgia-Eirini Deligiannidou, Rafail-Efraim Papadopoulos, Christos Kontogiorgis, Anastasia Detsi, Eugenia Bezirtzoglou, Theodoros Constantinides

**Affiliations:** 1 Laboratory of Hygiene and Environmental Protection, Department of Medicine, Democritus University of Thrace, 68100 Alexandroupolis, Greece; edeligia@med.duth.gr (G.-E.D.); paparafa@psy.auth.gr (R.-E.P.); empezirt@med.duth.gr (E.B.); tconstan@med.duth.gr (T.C.); 2 Laboratory of Organic Chemistry, School of Chemical Engineering, National Technical University of Athens, 15780 Athens, Greece; adetsi@chemeng.ntua.gr

**Keywords:** natural products, osteoarthritis, anti-inflammatory, cartilage degradation, antioxidant

## Abstract

The natural process of aging gradually causes changes in living organisms, leading to the deterioration of organs, tissues, and cells. In the case of osteoarthritis (OA), the degradation of cartilage is a result of both mechanical stress and biochemical factors. Natural products have already been evaluated for their potential role in the prevention and treatment of OA, providing a safe and effective adjunctive therapeutic approach. This review aimed to assess the therapeutic potential of natural products and their derivatives in osteoarthritis via a systematic search of literature after 2008, including in vitro, in vivo, ex vivo, and animal models, along with clinical trials and meta-analysis. Overall, 170 papers were obtained and screened. Here, we presented findings referring to the preventative and therapeutic potential of 17 natural products and 14 naturally occurring compounds, underlining, when available, the mechanisms implicated. The nature of OA calls to initially focus on the management of symptoms, and, in that context, several naturally occurring compounds have been utilized. Underlying a global need for more sustainable natural sources for treatment, the evidence supporting their chondroprotective potential is still building up. However, arriving at that kind of solution requires more clinical research, targeting the implications of long-term treatment, adverse effects, and epigenetic implications.

## 1. Introduction

In the words of the writer Janne Teller: “*From the moment we are born*, *we begin to die*.”; aging poses a natural, inevitable, and irreversible process. Even in a healthy organism, aging affects a broad spectrum of functioning aspects on physiological, cellular, and tissue level, which are subject to deterioration due to exposure to a series of environmental factors [1]. At a molecular level, this decline is characterized by the increasing accumulation of molecular damage due to free radicals environmentally and metabolically generated, errors and malfunctions in biochemical reactions caused without premeditation or external stimulus, and nutritional components. Changes in the synthesis and arrangement of key proteins, such as collagen, as well as proteoglycans over time, are observed due to aging as the metabolism of cells in articular joint tissues in normal and pathological conditions is highly affected [2,3]. 

It is a common belief that some reactive oxygen species (ROS) can cause or aggravate a series of human pathologies, mostly owed to direct damage induced to sensitive and biologically significant targets. Although the ability of antioxidants in the context of prevention or treatment of oxidative stress-related diseases and, therefore, their capacity to oppose the harmful effects of ROS is well documented, only a few antioxidants, including α-lipoic acid and some flavonoids, have found accepted clinical use [4,5]. Evidently, natural products or compounds thereof have the ability to oppose the activity of ROS due to their strong inhibitory and scavenging capacity of oxidative enzymes and free radicals; however, a deeper understanding of the mechanisms of action of those nutritional modulators in humans is still under investigation, with promising laboratory approaches, yet conflicting clinical results [6,7,8]. 

In the case of osteoarthritis (OA), the cartilage deterioration comes as a result of both biochemical factors, mainly matrix metalloproteinases (MMPs) and reactive oxygen species (ROS) and mechanical stress. Promising findings report that an increased intake of antioxidants may decrease free-radical damage to joint linings, which, in turn, diminish swelling and pain [9,10,11]. The communications of such findings have brought about the utilization of natural products and/or their novel structures and, in many cases, have led patients with chronic pain to turn to complementary or alternative medicine in addition to standard drug therapy [9,10,11]. 

Arthritis is a disease occurring almost in all age groups, while age-related osteoarthritis is the most recurrent form of joint disease in elder populations. However, the burden of OA is highly affected, as the elderly population increases, coupled with a growing prevalence of obesity [12,13]. Covering the spectrum from the management of obesity, which poses an important risk factor for OA, to the potential natural chondroprotective agents or nutrigenetic modulators, it is clear that nutrition can hold a key role in the prevention and management of OA [14,15]. International guidelines for the management of OA are based in three core interventions: weight control, general and joint-specific exercise, and education, while cases unaffected by these key interventions are often managed through non-pharmacological and surgical approaches, in the context of distressing OA, along with the use of analgesia and that favorably modify joint biomechanics [16].

It has already been documented via laboratory findings and real-world data that in the framework of common clinical observations in OA, agents, such as Interleukin-1 beta (IL-1β), interleukin-6 (IL-6), Phosphodiesterase_2_ (PDE_2_), and Tumor Necrosis Factor-alpha (TNF-α), which have an instrumental part in mediating the pathophysiological systems, as a result of synovial inflammation, are increasingly produced [17]. Additionally, subchondral bone may also hold an active role in the progress of OA as a source of biochemical modulators implicated in the OA pain course and cartilage deterioration [18,19,20]. Finally, the critical role of nuclear factor-κB (NF-κB) proteins that regulate the survival, stimulation, and differentiation of innate immune cells and inflammatory T cells is affected when stimulated by pro-inflammatory cytokines, stress-related factors, chemokines, and products of extracellular matrix (ECM) damage and stimulated molecules. This, in turn, triggers the expression of a series of genes that manifests as an increased collapse in the articular joint, leading to the onset and progression of the disease [18,19,20]. In this study, we presented an overview of the recent studies of natural products, which have been examined for the prevention and treatment of osteoarthritis in the setting of in vitro, in vivo, and ex vivo experiments or randomized clinical trials.

## 2. Materials and Methods 

For this review, the authors performed a systematic search of publicly available articles based on the criteria described in Table 1. 

Admissions in our review paper:

Note 1: Reviews will NOT be included in the analysis but will be useful for the introduction and/or be used to look for original papers.

Note 2: Population characteristics, General population: All genders (male/female), Age: adults (>18 years), Health status: Osteoarthritic patients (all concomitant diseases are included EXCEPT cancer in any form, lupus, fibromyalgia, osteoporosis, and psoriasis). Women during pregnancy and patients after replacement surgery are NOT included. 

Note 3: High tibial osteotomy, Knee arthroplasty, Articular cartilage repair, Knee replacement.

Note 4: Analgesics: acetaminophen, opioids (narcotics), and an atypical opioid called tramadol. Nonsteroidal anti-inflammatory drugs (NSAIDs): aspirin, ibuprofen, naproxen, and celecoxib. Corticosteroids. Hyaluronic acid.

Note 5: Western Ontario McMaster Universities osteoarthritis index (WOMAC), Visual analog scale, Lequesne's functional index as the commonly accepted methods to evaluate pain, stiffness, and physical function will be included.

All eligible papers were screened by two reviewers in terms of Title and Abstract relevance and then classified as per study design (articles were excluded in the stage of Title/Abstract screening due to intervention/comparator, population characteristics, study design). Following, the full text of included papers was screened by a third reviewer in case of conflicting opinions between the two initial reviewers, and the papers that met the inclusion criteria were distributed as per study design for data extraction (articles were excluded in the stage of full-text screening due to population characteristics, full text not available in English, and outcomes). 

After the selection and initial drafting of this review, all relevant papers were separated into one of two groups. The first group included published work referring directly to natural products or formulas of natural products, including mixtures, extracts, and any other forms assessing the effects of a naturally occurring product as a whole. The second group included published work referring to isolated natural compounds. Acknowledging that many of the beneficial properties for a series of natural products are the result of several components, or, in some cases, a single component existing in high concentration within a product, the results of this review were presented as parts of these two groups to properly emphasize the significance of each compound. Clinical trials of both groups were presented as the third part of this review.

## 3. Results

### 3.1. Natural Products and Formulas

#### 3.1.1. *Anthriscus* *sylvestris*

*Anthriscus sylvestris* (cow parsley or wild chervil) is widely distributed in Europe, Korea, and New Zealand as a common perennial herb [21]. Although *Anthriscus sylvestris*, family Apiaceae (*Umbelliferae*), is considered a noxious weed in certain states of America, research indicates that the leaves of this plant in the form of aqueous extract, or the essential oil of the plant, possess antioxidant, anti-inflammatory, and antibacterial properties [22,23,24]. More recently, the chondroprotective effects of the plant extract were investigated [21,25]. Notably, previous treatment with *A. sylvestris* leaves in the form of aqueous extract had inhibitory effects on Nitric Oxide (NO) and Prostaglandin E_2_ (PGE_2_) in the setting of secretion induced by lipopolysaccharide in RAW 264.7 cells and IL-1β-stimulated chondrocytes, without reporting cytotoxicity [21,25]. In both RAW 264.7 and human chondrocyte cell-lines, treatment with the plant extract dose-dependently attenuated the expression levels of inducible NO Synthase (iNOS), Cyclooxygenase-2 (COX-2), TNF- α, IL-1β, and IL-6 (RAW 264.6) [21]. Additionally, decreased expression levels were observed in MMP-3, MMP-13, and a disintegrin and metalloproteinase with thrombospondin type-4 motif (ADAMTS-4), as well as the inhibition of NF-κB activity in combination with decreased degeneration of aggrecan, collagen II, and proteoglycans in IL-1β-stimulated chondrocytes [25]. Finally, supplementation with the plant extract in different doses had up to 40% of inhibitory action against carrageenan-induced paw edema (related dose: 200 mg/kg of body weight) [21]. It is worth noting that previous research has highlighted the anti-inflammatory potential of deoxypodophyllotoxin, which has been identified as a known naturally occurring flavolignan of the plant [26]. On that note, the effects of the aqueous extract (or other fragmentations) against iNOS, cyclooxygenases, or metalloproteinases expression or active pathways implicated in inflammation [27,28] can very well be attributed to a single compound, which is a common theory for several natural products and formulations.

#### 3.1.2. Avocado/Soybean

Avocado/soybean unsaponifiables belong in the class of symptomatic slow-acting drugs, which following published reports, have demonstrated the potential to lessen the synthesis of some mediators of osteoarthritis-related inflammation by interfering with a variety of molecules and pathways implicated and cartilage catabolism, in addition to demonstrated pain alleviation and physical function improvement [29,30]. In the context of anticatabolic features of Avocado/soybean unsaponifiables (the oily extracts that remain after soap manufacture, which include phytosterols, lipophilic vitamins, and tri-terpenoids), the administration of the extract exhibited the potential to avert cartilage degradation by suppressive means over the expression and activity of biomarkers related to non-collagenous proteins, matrix metalloproteinases (MMP-3, MMP-13), and increasing Tissue Inhibitor of Metalloproteinase (TIMP-1) [31]. Namely, the findings of animal studies conducted over the last decade indicate that intervention was able to reduce the joint edema resulting from Monosodium Iodoacetate (MIA) injection after 30 days of intervention (dose: 35 mg/day) [29,32], while, in treated dogs, researchers observed histological improvement of cartridge lesions and significant abatement in iNOS and MMP-13 levels [33]. Finally, the joint diameter was decreased in a moderate yet statistically significant way, and Hindlimb weight-bearing notably improved in the treatment group [29,32]. The finding reflects the capacity of this treatment to temper painful OA in the in vivo setting, which is also observed via clinical trials that have been discussed further in this review. 

#### 3.1.3. *Chrysanthemum* *zawadskii*

*Chrysanthemum L*. (Asteraceae-Anthemideae) is a plant variety widely found in East Asia. Published reports have documented the anti-inflammatory perspective and antioxidant capacity of the plant extract in the setting of in vitro and in vivo studies [34,35,36], with the experimental focus recently targeting the inhibitory assets on the differentiation of osteoclasts and protective activities against arthritis [37]. Evidently, pro-inflammatory biomarkers—IL-1β, IL-6, COX-2, and iNOS—were inhibited by actions of a hexane/ethanol extract (or other fragmentations) of *Chrysanthemum zawadskii* in RAW 264.7 cells, potentially through heme oxygenase-1 induction [38,39]. Furthermore, the ethanol extract of Chrysanthemum exhibited suppressive attributes over MMP-2 and MMP-13 levels in MIA-induced osteoarthritic rats [40]. Additionally, oral intervention with 50–200 mg/kg *Chrysanthemum zawadskii* was found to suppress the protein expression of pro-apoptotic molecules and to attenuate serum cartilage oligomeric matrix protein [40]. Finally, improvement in common OA symptoms of swelling and walking difficulty was also reported after oral administration to rats of different concentrations: 50, 100, and 200 mg/kg [40]. An intriguing finding from a recent study of 2016 has also highlighted the anti-adipogenic qualities of the *Chrysanthemum zawadskii* extract [41], studied among other herbs, providing yet another possibility of the plant within the framework of OA management and/or prevention.

#### 3.1.4. Guilu Erxian Glue

There is a growing interest in an herbal mixture that originated in Chinese tradition with reported useful assets in the treatment of some conditions. In particular terms, guilu erxian (GE) glue has been under investigation for its therapeutic potential against joint pain, muscle strength, and disease progression within the context of knee OA, in addition to bone marrow injury and osteoporosis, mostly in the cellular level [42,43,44,45]. For the purposes of this review, we presented the findings of a 2018 study by Chou et al., who evaluated two formulations extracted from GE glue, namely, GE paste and GE liquid in male-induced osteoarthritic mice [44], and reports of a 2014 clinical trial in a population of elderly men with knee OA, which have been presented in the respected segment of the review [45]. In the in vivo setting of anterior cruciate ligament transection (ACLT)-induced OA, the effects of a 28-days intervention with GE in the form of either paste or liquid were evaluated in two treatment doses (100 mg/kg/day or 300 mg/kg/day) [42] against phosphate-buffered saline and celecoxib (10 mg/kg/day). Notably, the intervention with the liquid mixture formulation in the minimum dose was able to considerably lessen the mRNA levels of IL-1β, IL-6, and TNF-α, as well as the protein expression of IL-1β and TNF-α, compared to either formulation at high dose and celecoxib [44]. Both formulations presented a notable and dose-dependent reduction of osteolytic lesions, cartilage erosion, and bone spur formation in addition to reductions observed in proteoglycan, chondrocyte, and cartilage damage [44]. Although the formulations of this mixture presented chondroprotective properties with anti-inflammatory characteristics, the evidence supporting those allocations remain inconclusive. 

#### 3.1.5. Japanese Pepper or Korean Pepper (*Zanthoxylum piperitum*)

*Zanthoxylum piperitum* (Rutaceae family)—an aromatic spiny shrub—is commonly known as Japanese pepper or Korean pepper and has also been reported to have antibacterial, antimicrobial, and some anti-inflammatory attributes, mostly investigated in the form of essential oil [46,47,48,49]. *Zanthoxylum piperitum* essential oil was tested in an animal model setting. The outcomes of the 2016 study demonstrated some remarkable primary effects on the first phase of the formalin-induced licking, glutamate, and hot plate tests; however, these positive conclusions did not endure throughout the study as the oil did not affect reducing paw licking induced by capsaicin or against inflammation induced by carrageenan, as observed through further testing in the same study [50]. 

In a more recent in vitro study investigating *Zanthoxylum piperitum* ethanolic extract, the findings revealed strong inhibitory activity of the extract in the Lipopolysaccharide (LPS)-induced RAW 264.7 cell line, as well as suppressive characteristics against reactive oxygen species production, COX-2, and iNOS. Further, in vivo analysis on the MIA-induced OA model revealed that 100 mg/kg oral-treatment significantly inhibited MIA-induced edema and articular cartilage thickness. Notably, acetic acid, heat, and formalin-induced pain were remarkably decreased by both intervention doses of the ethanolic plant extract (50 and 100 mg/kg) in a dose-dependent manner. Finally, the inflammation and pain-related effects of the intervention were also evaluated in BV-2 cells and by mouse-ear thickness and biopsy punch weight measurements [51]. 

#### 3.1.6. *Litsea* *japonica*

The qualities of Litsea (genus of the Laurel family, *Lauraceae*), mostly in the form of the ethanolic (EtOH) or hexane fragmentations of the plant, as an antioxidant agent with anti-inflammatory characteristics, have been evaluated, inter alia, within the setting of OA [52,53,54,55,56,57,58]. Data reported from in vitro research, investigating the anti-inflammatory assets of a variety of fractions (EtOH or other) of this plant within the context of lipopolysaccharide (LPS)-stimulated RAW 264.7 cells, suggest a considerable inhibitory activity of the intervention on modulators of inflammation (NO and PGE_2_, iNOS, and COX-2), expression levels, along with pro-inflammatory cytokines, IL-1β, IL-6, TNF-α, and NF-κB (p65 and p50) activation, and mitogen-activated protein kinase (MAPK) phosphorylation [52,53,54].

According to the 2015 in vivo study, treatment with a 70% ethanolic extract of *Litsea japonica* fruit in MIA-induced OA rats was found to improve the bone volume and cross-section thickness, while it also curbed the expression levels of inflammatory cytokines. It must also be noted that high doses (100 mg/kg or 200 mg/kg) in the treatment groups resulted in a remarkable 80% inhibition of MMPs or MMPs tissue inhibitors threshold when compared to the respected control group [55]. Similar results were reported in the 2017 in vivo study, in which the n-hexane extract of *Litsea japonica* fruit flesh considerably reduced the difference in weight-bearing capabilities of the hind paws among healthy and MIA-treated rats, while cartilage and bone destruction were also hindered [56]. Additionally, this treatment actively attenuated MMP-2, MMP-9, COX-2 expression, along with serum levels of deoxypyridinoline (DPD) and osteocalcin. Moreover, this intervention considerably suppressed inflammatory cells’ infiltration and attenuated TNF- α, IL-1, and IL-6 levels in joints. Accordingly, the intervention also lessened NO, PGE_2_, IL-6, and TNF- α production in LPS-activated macrophages, while serum concentrations of leukotriene B4 (LTB4) and lipoxygenase (LOX) were also essentially lowered [56]. Finally, it must be noted that, in some cases, the curative assets of the extract/or extract fractions, evaluated in RAW 264.7 cell-line, have, in fact, been owed to the single action of isolated constituents, namely, litsenolide-B_2_, which has exhibited useful properties against pro-inflammatory mediators [57].

#### 3.1.7. Mistletoe Fig (*Ficus deltoidea*)

*Ficus deltoidea* Jack, commonly known as mistletoe fig, found in several parts of the globe, has been traditionally utilized in folk medicine. Several studies have determined some qualities of the plant extract, in the context of antioxidant or antimicrobial competence, while others have focused on its inhibitory assets against inflammation [59,60,61,62]. Three studies that took place over the past four years have assessed the potential of the plant in the context of experimental models [63,64,65]. Initial in vitro results of the 2016 survey investigating the impact of *Ficus deltoidea* (FD) leaves extract supplementation on OA through IL-1β-induced bovine cartilage explant exhibited inhibitory properties of this intervention on proteoglycan loss in addition to an increasing tendency of chondrocytes proliferation [63]. It must be noted that this research studied the supplementation with the plant extract against diclofenac, in the setting of MIA-induced OA postmenopausal rats. Data reported from this 28-days intervention in healthy or osteoarthritic rats suggested that levels of IL-1β, PGE_2_, Procollagen II N-terminal pro-peptide (PIINP), and C-telopeptide of type II collagen (CTX-II) elevated in the serum OA rats, and largely reduced after supplementation with the FD extract and the active comparator [63]. 

Both 2016 and 2017 studies, referring to MIA-induced OA postmenopausal rat models, demonstrated the effects of a decrease in IL-1β, PGE_2_, and C-telopeptide type II collagen levels in serum, while observations of attenuated cartilage erosion were reported in both intervention groups. However, the second (2017) research also highlighted that similarly to diclofenac, the plant extract intervention (dose equivalent of 60 mg/kg for humans) down-regulated the IL-1β, PGE_2_ receptor, and MMP-1 mRNA expressions in the osteoarthritic cartilages, which seemed to be dose-dependent [64]. Finally, a study conducted in 2018 by the same team aimed to provide bases on the inconclusive osteoporosis/osteoarthritis relationship [65].

#### 3.1.8. Pink Trumpet Tree – (*Tabebuia avellanedae*)

*Handroanthus impetiginosus* or *Tabebuia avellanedae* or pink trumpet tree is a native Bignoniaceae tree of America; however, different varieties of the tree may have also been introduced to other locations. Several studies have documented a series of characteristics of the plant, derived from various locations (Paraguay, Brazil, India, and others), using ethanolic, methanolic, or water extracts of mostly the bark of the plant [66,67,68,69]. More commonly, the antimicrobial properties of the plant have been investigated; however, there are some reports of anti-inflammatory activity [70,71,72,73].

In an early 2008 study, evaluating the anti-inflammatory properties of the water extract of *Tabebuia avellanedae* in the setting of lipopolysaccharide-stimulated macrophages, results suggested that the treatment had significant inhibitory effects on PGE_2_ and NO production, as well as the expression of COX-2 and iNOS, in LPS-stimulated RAW 264.7 cells [70]. Similarly, in the setting of induced edema at the mouse ear by arachidonic acid (COX-2 activator) or croton oil (LOX activator), a week’s supplementation with the water extract of *Tabebuia avellanedae* (100 mg/kg) was able to alleviate the arachidonic acid-induced edema as opposed to the edema induced by croton oil, which was not actively affected [70]. In a similar context, the ethanolic extract (doses: 100 mg/kg or 200 mg/kg) was evaluated against pain and inflammation versus a control group [71]. The results of this study demonstrated an approximate 30% increase in pain in the treatment group at dose 200 mg/kg. The same dose was additionally able to inhibit 30%–50% of inflammation of tissue plasminogen activator (tPA)-induced paw edema, (arachidonic acid or carrageenan) compared to control [71]. Finally, it must be said that TabetriTM had also documented inhibitory effects on inflammatory mediators and proinflammatory cytokines in a dose-dependent manner. Although this product is referred to as the ethanol extract of *Tabebuia avellanedae*, as a commercial product, it did not fall in the scope of this review and was not further investigated [74]. 

As in the cases of many other plant extracts exhibiting therapeutic characteristics, it must be noted that a number of compounds isolated from the water extract of the plant, inter alia, some cyclopentenyl esters, and the known naphthoquinone: lapachol, have been documented to have in vitro anti-inflammatory properties as well [72,73,75]. Notably, some of these derivatives effectively ameliorated NO, PGE_2_, TNF-α, and IL-1β production. On that note, it is essential to highlight once more that research has yet been inconclusive on whether the plant extract characteristics are owed to some selected compounds or its combined properties.

#### 3.1.9. Pomegranate (*Punica granatum*)

**Scheme 1:**
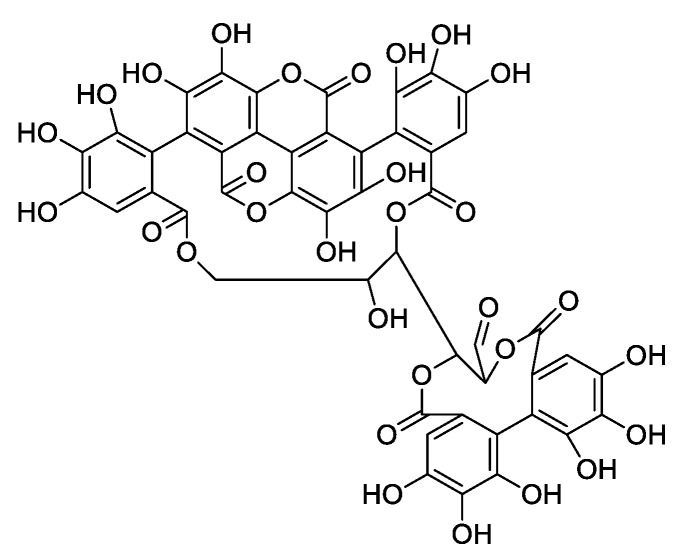
Punicalagin structure.

Pomegranate (*Punica granatum* L.) is undoubtedly one of the oldest known edible fruits of the Punicaceae family and has long been studied for its effects on a number of diseases [76,77,78]. A recent 2018 study investigated the anti-inflammatory properties of pomegranate peels in the form of their acetone extract and reported significant reductions of iNOS, COX-2, and MMP-13 in primary rat chondrocytes, as well as changes in the weight-bearing ratio in experimental OA in rats [79]. The same study has further evaluated the properties of punicalagin (Scheme 1). This ellagitannin also exhibited some chondroprotective properties after 28 days of supplementation (0.50 mg/kg), but it was not superior to the peel extract against PGE_2_ production (19.1%) [79]. Almost a decade earlier, a study was also conducted in an animal model using different doses of pomegranate juice (4 mL/kg, 10 mL/kg, or 20 mL/kg) in the setting of early MIA-induced OA. As reported, although proteoglycan and cell proliferation were not actively affected, the intervention (dose-dependently) prevented the negative effects of iodoacetate, as well as chondrocyte damage [80]. In a similar way, a 2010 study investigating the potentials of a polyphenol-rich pomegranate extract in the context of IL-1β-induced human chondrocytes demonstrated that the intervention was able to actively decrease the IL-1β-induced activation of mitogen-activated protein kinase kinase-3 (MKK3), p38α-MAPK isoform, and DNA binding activity of the transcription factor Runt-related transcription factor 2 (RUNX-2) [81].

#### 3.1.10. Ryupunghwan

**Scheme 2:**
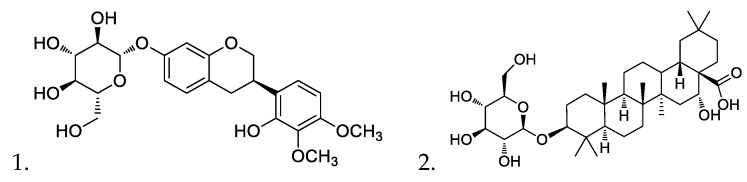
Structures of isomucronulatol 7-O-β-d-glucoside (**1**) and Ecliptasaponin A (**2**).

In a recent study, Hong G.U. et al. presented the OA modulating potential of a botanical formula, which is particularly common in Korea, mostly well known as Ryupunghwan (RPH) [82]. The formula contains a number of natural products, such as *Astragalus membranaceus*, *Turnera diffusa*, *Achyranthes bidentata*, *Angelica gigas*, *Eclipta prostrata*, *Eucommia ulmoides*, and *Ilex paraguariensis*, most of which are traditionally used in folk medicine. The formula was tested in the context of positive properties against OA, via in vitro experiments on cellular IL-1β-stimulated chondrosarcoma, SW1353 cell model [82]. As reported in this recent work, the pre-treatment with RPH had a suppressive effect on the mediators that contributed to the progression of osteoarthritis by inhibiting collagen 2, COX-1-2, MMP-13, TNF-α, and IL-13 expression [82]. In the case of RPH formula, two components—isomucronulatol 7-O-β-d-glucoside and Ecliptasaponin A (Scheme 2)—were found to be present in higher concentration levels, and Ecliptasaponin A was further found to play a crucial role against OA, even higher than isomucronulatol 7-O-β-d-glucoside (IMG’s) [82].

#### 3.1.11. *Sargassum* *serratifolium*

**Scheme 3:**
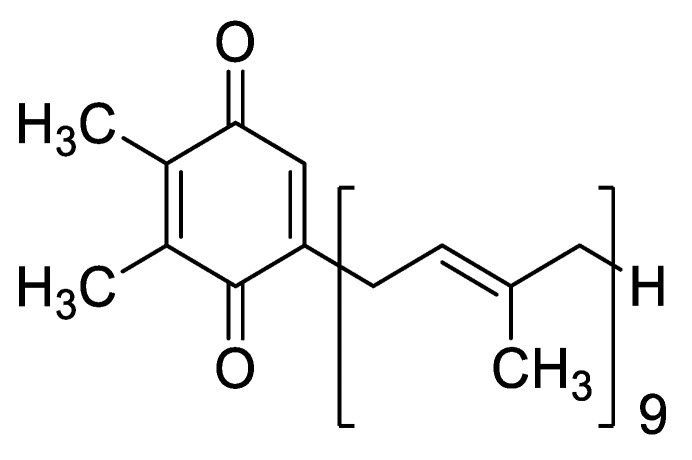
Plastoquinone structure.

*Sargassum* spp. (a brown seaweed) and plastoquinones (Scheme 3), which are present in high volumes in *Sargassum serratifolium* (C. Agardh), are known for several documented substantial anti-inflammatory and anti-oxidative assets [83,84]. Notably, sargachromenol, sargahydroquinoic acid, and sargaquinoic acid have been identified in a 2017 study presented by Joung E. et al. as the main components of inflammatory suppression in the ethanolic fraction of the plant, repressing NO production [85]. A recent 2018 research by Park C. et al. investigated *Sargassum serratifolium* extract (EtOH 70%) in human-stimulated IL-1β chondrocyte cell line and early murine synovial cells [86]. The high antioxidant profile of the EtOH fragment was found in both cell lines. as well as active reduction of ROS, while anti-inflammatory assets were also documented as a result of the decreased expression of COX-2, iNOS, in addition to decreased PGE_2_ and NO production, after supplementation [86]. Additionally, this Sargassum EtOH fragment intervention down-regulated MMP-1, -3, and -13 gene expression in SW1353 IL-1β-treated chondrocytes, while the NF-κB and p38 MAPK kinase activities were also affected [86]. It is worth noting that similar results were reported by Joung E. et al. in a cellular setting on pro-inflammatory cytokines in mouse serum [85].

#### 3.1.12. Schisandrae Fructus (*Schisandra chinensis* (Turcz.) Baill)

*Schisandrae Fructus* (SF), the dried fruit of *Schisandra chinensis* (Turcz.) Baill. (Magnoliaceae), is another natural product, traditionally known in the Chinese culture, but also popular in the Russian culture, yet widely used as a folk remedy for the prevention and management of several chronic inflammatory diseases [87]. SF extract in the setting of IL-1β-stimulated SW1353 chondrocytes actively attenuated IL-1β-induced expression of matrix MMP-1, -3, and -13, while the intervention also reduced the elevated levels of COX-2 and iNOS associated with PGE_2_ and NO production [88]. In addition, a 2015 study showed that SF markedly suppressed the nuclear translocation of NF-κB by blocking inhibitor κB-alpha degradation and exhibited repressive properties of c-Jun N-terminal kinase and p38 MAPK phosphorylation [88].

### 3.2. Pure Natural Compounds 

**Scheme 4:**
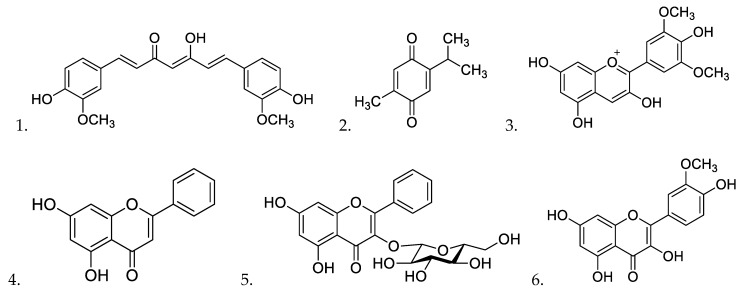

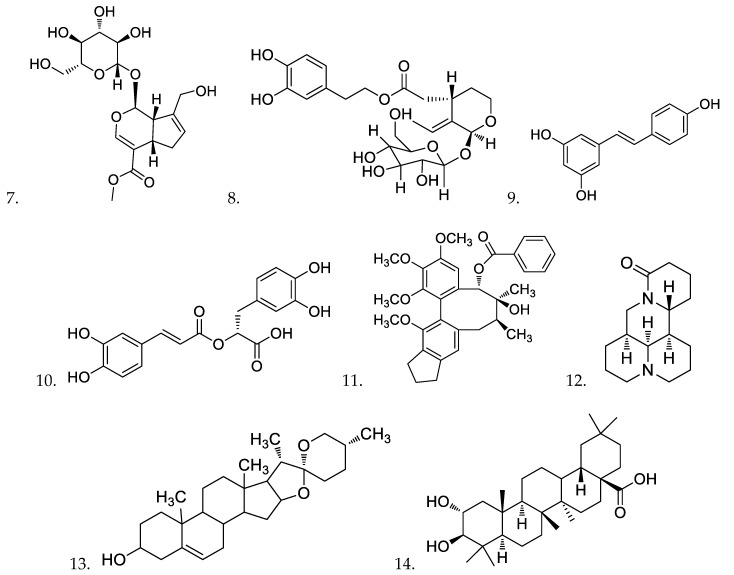
Structures of natural compounds evaluated for their potential in the context of osteoarthritis (OA). **1**: Curcumin; **2**: Thymoquinone; **3**: Malvinidin; **4**: Chrysin; **5**: Astragalin; **6**: Isorhamnetin; **7**: Geniposide; **8**: Oleuropein; **9**: Resveratrol; **10**: Rosmarinic Acid; **11**: Schisantherin A; **12**: Matrine; **13**: Diosgenin; **14**: Maslinic acid.

Over the last decade, numerous compounds isolated from natural products have been investigated in the context of inhibiting inflammation in osteoarthritis. Here, we presented a summary of these findings, mostly based on research conducted in chondrocytes and some OA animal models. Among others, compounds like curcumin, also known as turmeric, with well-documented effects against some conditions, including arthritis [89,90,91]; thymoquinone, a compound isolated from fennel flower (*Nigella sativa*); representatives of flavonoids (anthocyanins (malvinidin), flavones (chrysin), and flavonols (astragalin and isorhamnetin); geniposide, an iridoid glycoside, with some affiliations against a variety of chronic inflammatory conditions [92,93]; the secoiridoid glycoside oleuropein; the well-known bioactive compound resveratrol, with many biological properties [94] and specific inhibitory properties of NF-kB, as well as COX-2 gene expression and enzyme activity [95], and the equally known rosmarinic acid with also numerous therapeutic assets [96] are included in this review. The chemical structures of all the compounds investigated in this review regarding their anti-inflammatory and chondroprotective potential in the context of OA are presented in Scheme 4, while the main effects and research characteristics are summarized in Table 2.

#### 3.2.1. Curcumin

Curcumin (Scheme 4), also known as turmeric, is a compound with potential protective effects, i.e., against cancer, Alzheimer's disease, heart failure, diabetes, and arthritis [89,90,91]. Focusing on gene expression profiling in human T98G neuroglia cells, a 2018 study showed that supplementation with curcumin and with curcumin-Boswellia serrata combination formula selectively down-regulated opioid-related nociceptin receptor 1 (OPRL1) expression (5.9-fold and 7.2-fold, respectively), which, according to this study, inhibited nociceptin opioid peptide (NOP) production. Further findings of these interventions suggest an essential reduction of neuropathic pain, neuroinflammation, and suppressed a disintegrin and metalloproteinase (ADAM) metallopeptidase gene ADAMTS-5 expression (11.2-fold and 13.5-fold, respectively) [97]. Similar outcomes were observed after supplementations with a ginger/curcumin combination in MIA-induced OA rats. In this setting, administration doses of 200 mg/kg or 400 mg/kg resulted in a notable decrease in serum levels of cartilage oligomeric matrix protein (COMP), hyaluronic acid (HA), myeloperoxidase (MPO), malondialdehyde (MDA), and IL-1beta and particularly higher superoxide dismutase (SOD) levels (*p* < 0.0001) [98]. 

#### 3.2.2. Geniposide

Geniposide (Scheme 4), an iridoid glycoside purified from fruits (most well-known *Gardenia jasminoides*) and herbs, has been commonly used in folk medicine for many chronic inflammatory conditions [92,93]. A 2018 study by Chen et al. demonstrated that in the context of a surgically-induced OA rabbit model, this compound effectively suppressed IL-1, TNF- α, NO, and MMP-13 expression in animals synovial fluid [99]. Similar in vitro and in vivo results were obtained by Pan T. et al. as the intervention promoted the production of collagen II and decreased the expression of proapoptotic molecules—Bax, Cyto-c, C-caspase 3—as opposed to an increasing Bcl-2 expression [100].

#### 3.2.3. Oleuropein 

Oleuropein (Scheme 4), a secoiridoid glycoside that is the methyl ester of 3,4-dihydro-2H-pyran-5-carboxylic acid, is considered as the most prevalent phenolic component in olive leaves and seeds, pulp and peel of unripe olives with potent anti-inflammatory effects. As reported by an early 2017 study, oleuropein treatment (doses: 10 μΜ, 50 μΜ, and 100 μM) on human OA chondrocytes largely inhibited the IL-1β-induced production of NO, PGE_2_, COX-2, iNOS, MMP-1, MMP-13, and ADAMTS-5. Additional findings have introduced the preventative characteristics of this compound against the degradation of aggrecan and collagen-II, as well as inhibitory effects on NF-κB and MAPK activation, suggesting that potential therapeutic effect of oleuropein on OA is due to its effect on those signaling pathways [101]. 

#### 3.2.4. Thymoquinone

Non-inferior results are reported for a lesser-known, yet commonly used medicinal plant, *Nigella sativa* (N. *Sativa*) (Family *Ranunculaceae*) [102] and thymoquinone (Scheme 4); it is a most prominent compound with reported hepatoprotective, anti-inflammatory, anti-cancer, and antitumor effects [103]. A 2013 study reported that thymoquinone concentration-dependently inhibited IL-1β-induced COX-2, MMPs, iNOS, NO, and PGE_2_ production, as well as IL-1β-induced NF-κB and MAPKs activation and inhibitor of kappa B (IκBα) degradation, in the setting of IL-1β-stimulated human OA chondrocytes [104]. 

#### 3.2.5. Flavonoids

Representatives of flavonoids (anthocyanins - malvinidin), flavones (chrysin), and flavonols (astragalin and isorhamnetin) have also been studied in the context of OA. Namely, the main anthocyanins found in three Thai purple rice cultivars (mostly found as 3-glucosides of peonidin and cyanidin) attenuated the inhibition of porcine cartilage degradation in an experimental model, as well as the induction of MMPs caused by IL-1β-stimulated human chondrocytes. Notably, the effects observed to be anthocyanin concentration-dependent, while protocatechuic acid, anthocyanin metabolite, exhibited chondroprotective potential by reducing glycosaminoglycans and collagen (COL) breakdown in IL-1β/ Oncostatin M (OSM)-induced porcine cartilage explants [105].

Malvidin (Scheme 4) (one of the most widespread anthocyanidins) exhibited significant pain-relieving effects in MIA-induced OA Wistar rats and decreased the expression level of apoptotic marker senescence-associated beta-galactosidase (SA-beta-gal) in chondrocytes. It is worth noting that this treatment remarkably reversed the up-regulated expressions of IL-1β, IL-6, TNF-α, and MMPs in cartilage tissues. Furthermore, malvidin suppressed the NF-kB pathway via an NF-kB inhibitor (IκBalpha)-independent manner through decreasing p65 nuclear transportation [106]. 

Chrysin (Scheme 4), a natural flavonoid extracted from honey and propolis, has been reported to have anti-inflammatory properties. The intervention on human OA chondrocytes pre-treated with chrysin (10, 50, and 100 μM) for 2 hours and subsequently with IL-1β for 24 hours significantly inhibited the IL-1beta-induced production of NO and PGE_2_; expression of COX-2, iNOS, MMP-1, MMP-3, MMP-13, ADAMTS-4, and ADAMTS-5; and degradation of aggrecan and collagen-II. Furthermore, chrysin notably suppressed IL-1beta-stimulated IκB-alpha degradation and NF-κB activation [107]. 

Astragalin (Scheme 4), a bioactive compound found in *Rosa agrestis*, demonstrated anti-inflammatory properties in IL-1β-stimulated human OA chondrocytes. The outcomes of a 2015 study demonstrated that astragalin was able to dose-dependently inhibit IL-1β-induced NO and PGE_2_ production, as well as iNOS and COX-2 expression, IL-1β-induced NF-κB and MAPK activation in human OA chondrocyte, by activating the peroxisome proliferator-activated receptor gamma (PPAR-γ) [108]. Similar effects were reported for isorhamnetin (Scheme 4)—a flavonoid mainly derived from the fruit of *Hippophae rhamnoides L* [109]. Pre-treatment with this compound actively suppressed the expression of stromelysin-1 and collagenase-3, NO, PGE_2_, iNOS, and prostaglandin G/H synthase 2 in chondrocytes. Furthermore, this treatment, as seen in astragalin, also inhibited the expression of NF-κB and transcription factor p65 [109].

#### 3.2.6. Resveratrol

Polyphenols have been extensively investigated concerning their antioxidant, anti-inflammatory, and immunomodulant properties in many inflammatory chronic conditions. Resveratrol (Scheme 4) is a phytoalexin found in particularly high concentrations in grape skin and red wine. Notably, red wine is a widely consumed beverage with many biological properties, including protective effects against oral infections and related bone (osteoarthritis, osteomyelitis, periprosthetic joint infections) and cardiovascular diseases [94].

Resveratrol is, among the bioactive compounds present in wine, the most well studied for its strong and specific inhibitory properties on NF-kB, as well as COX-2 gene expression and enzyme activity [95]. Early 2008–09 studies reported that resveratrol, like N-Ac-Leu-Leu-norleucinal (ALLN), suppressed IL-1β-induced proteasome function and the degradation of IκBα without affecting IκBα kinase activation, IκBα-phosphorylation, or IκBα-ubiquitination. Finally, there is also evidence for resveratrol’s suppressing ability on IL-1β-induced apoptosis, caspase-3 activation, and PARP cleavage in human articular chondrocytes [110,111,112].

A 2017 study aimed to evaluate the biological effects of resveratrol implicated in human chondrocytes stimulated with IL-1β involving both dependent and independent toll-like receptor-4/myeloid differentiation primary response 88 (TLR4/MyD88) signaling pathways. Co-treatment with resveratrol was able to reduce the cell viability of IL-1β-stimulated chondrocytes. Besides, while IL-1β stimulation increased the upregulation of TLR4 and downstream targets in both dependent and independent TLR4/MyD88 signaling pathways, co-treatment with resveratrol suppressed both catabolic and inflammatory responses in a dose-dependent manner. Resveratrol reduced the expression levels of MMP-13, IL-6, TNF-α, phospho-interleukin-1 receptor-associated kinase 4 (p-IRAK4), and TLR4- tumor necrosis factor receptor-associated factor 6 (TRAF6) mediators of the TLR4/MyD88 signaling pathways [113]. Similar results were observed in obesity-related OA in an animal model. The overall outcomes of this 2017 study indicate that high-fat diet-induced obesity could trigger the onset of OA, and resveratrol might alleviate OA pathology by decreasing systematic inflammation and/or inhibiting the TLR4 signaling pathway in cartilage, posing as a potential therapeutic approach for obesity-related OA [114]. Limagne E. et al. investigated the pro-inflammatory paracrine interactions between human primary chondrocytes and macrophages following IL-1β treatment. Resveratrol showed a strong inhibitory effect on the pro-inflammatory marker secretion, which was dependent on NFκB inhibition in the chondrocytes [115].

The accumulation of advanced glycation end products (AGEs) in joints contributes to the pathogenesis of cartilage damage in OA. In the setting of AGEs-stimulated porcine chondrocytes and cartilage explants, a 2010 study showed that AGEs-induced expression of iNOS and COX-2 and production of NO and PGE_2_ were suppressed by resveratrol. This study suggests that the effects of this treatment were likely mediated through inhibiting IKK-IκBα-NF-κB and c-Jun N-terminal kinase/Extracellular signal-regulated kinases-Activated protein-1 (JNK/ERK-AP-1) signaling pathways. By targeting these critical signaling pathways, resveratrol decreased AGEs-stimulated expression and activity of MMP-13 and prevented AGEs-mediated destruction of collagen II. Histochemistry analysis further confirmed that resveratrol could prevent AGEs-induced degradation of proteoglycan and aggrecan in cartilage explants, providing additional evidence to the potential therapeutic benefit of resveratrol in the treatment of OA [116].

#### 3.2.7. Rosmarinic Acid

Rosmarinic acid (Scheme 4), most well known for being the active ingredient in Rosemary, presents a number of interesting biological activities, e.g., antiviral, antibacterial, anti-inflammatory, and antioxidant [96]. Reports from a 2017 study suggest that rosmarinic acid increased type II collagen, sulfated-proteoglycan, COX-2, and PGE_2_ production in a dose- and time-dependent manner in rabbit articular chondrocytes while suppressing the expression of MMP-13. The treatment also activated extracellular signal-regulated kinase (ERK)-1/2 and p38 kinase signaling pathways [117]. Similar results were observed in rat IL-1β-stimulated chondrocytes, where IL-6 production was inhibited, as well as the gene and protein expression of ADAMTS-4, ADAMTS-5, aggrecan (ACAN), and type II collagen, overall suggesting, as previously observed, that rosmarinic acid could inhibit extracellular matrix (ECM) degradation in OA [118].

#### 3.2.8. Other Phenolics

Schisantherin A (SchA) (Scheme 4) is a dibenzocyclooctadiene lignan isolated from the fruit of *Schisandra sphenanthera*. In the setting of IL-1β-induced OA chondrocytes, treatment with this compound significantly inhibited NO, PGE_2_, and TNF-α production in a dose-dependent manner. Moreover, IL-1β-induced MMP-1, -3, and -13 expression and NF-κB and MAPKs activation were essentially suppressed [119]. Similar results were observed in the case of diosgenin (Scheme 4), which is a steroidal saponin found in several plants, including *Solanum* and *Dioscorea* species. Diosgenin was found able to additionally inhibit the expression of COX-2 in human OA chondrocytes and suppress the degradation of IκB-α [120]. Additionally, MMP-3 and MMP-13 expression, phosphorylation of p-38, extracellular regulated kinase (ERK), c-Jun-N-terminal kinase (JNK), and IκB-α degradation induced by IL-1beta in chondrocytes were significantly curbed by Matrine, an alkaloid found in plants from the genus *Sophora*. This compound, which also acts as a κ-opioid and μ-opioid receptor agonist, significantly inhibited the IL-1β-induced apoptosis in chondrocytes, while it increased the production of TIMP-1 [121].

### 3.3. Clinical Trials

#### 3.3.1. *Acacia catechu* and *Mori folium* Standardized Blend (UB1306)

Acacia catechu (*Mimosa* family) extracts and *Mori folium*, the leaves of *Morus alba L*., have been traditionally used as a dietary supplement or folk medicine due to the documented anti-inflammatory activity, as well as protection of the liver and hypertension, respectively [122]. The aqueous extract of *Acacia catechu* heartwood is a good source of catechin and epicatechin, with smaller quantities of flavonoids, which have well-documented health benefits [123], also in the context of OA as it has been observed in this review. In a randomized, double-blinded, placebo-controlled, parallel design, 135 subjects received UP1306 (a standardized blend of Acacia catechu and Mori folium), glucosamine chondroitin, or placebo for 12 weeks in an effort to determine the effects of UP1306 on discomfort and function in adults with OA of the knee. The outcomes displayed an improvement in discomfort, stiffness, and daily activity (WOMAC questionnaire and Visual Analog Scale (VAS) score) within all groups. Namely, the Western Ontario McMaster universities osteoarthritis index (WOMAC)-pain sub-score was decreased by 51% for UP1306, by 45% for glucosamine chondroitin, and by 46% for placebo. Although there were no changes in TNF-α levels, a notable difference in urinary C-telopeptides of type II collagen (CTX-II), a marker of cartilage degradation, was observed after 12 weeks (*p* = 0.029). Notably, serious adverse events (AEs) were not observed in this study. In a sub-group of 30 of the 133 subjects in the safety population, 43 clinically non-significant adverse events were observed (15 in the UP1306 group, 10 in the glucosamine and chondroitin group, and 18 in the placebo group), while only 14 events were considered to be related to the study product [124].

#### 3.3.2. Coriander, Litsea Japonica, and Guilu Erxian Jiao

Coriander (*Coriandrum sativum L*.), a member of the *Apiaceae* family, is among the most widely used medicinal plants, possessing nutritional and medicinal properties [125]. Treatment with coriander (*C. sativum L*.) leaf powder (5 g/day for 60 days) on OA patients suppressed LOX and catalase activity (CAT) in erythrocytes as well as alkaline phosphatase activity and erythrocyte sedimentation rate (ESR) while improving serum β-carotene, vitamin C, and calcium levels. In addition, increased activities of glutathione-S-transferase (GST) and decreased glutathione (GSH) content were also reported in the treated OA patients [126]. 

#### 3.3.3. Guilu Erxian Jiao

The initial focus in osteoarthritis is the management of the painful symptoms aside from biochemical markers assessment, and, to that end, natural products, such as guilu erxian jiao, have also been evaluated. Elderly men were included in a 2014 clinical trial in an effort to evaluate the effects of guilu erxian jiao in the context of knee osteoarthritis (KOA). This 12-week study demonstrated overall significant increases in muscle strength and knee flexibility, along with limited pain (*p* < 0.01) in the intervention group (n = 21) [45]. 

#### 3.3.4. *Litsea* *Japonica*

Litsea is a genus of the Laurel family, Lauraceae, including numerous accepted species found in tropical and subtropical areas. Regarding the efficacy context of this plant (extract) (using low-dose group; dose: 100 mg/d and high-dose group; dose: 200 mg/d), the record showed that WOMAC scores on the pain (*p* = 0.0293), stiffness (*p* = 0.0002), and function (*p* = 0.0152) subscales significantly improved during a 12-weeks randomized, double-blind, placebo-controlled study, involving 87 OA knee patients, underlining dose-dependency of the outcomes [58].

#### 3.3.5. Maslinic Acid 

Maslinic acid (MA) (Scheme 4) is a pentacyclic triterpene with a range of biological activities, namely, antitumor, antidiabetic, antioxidant, cardioprotective, neuroprotective, antiparasitic, and growth-stimulating [127]. In a recent 2018 study, Yoon et al. investigated the possible synergetic effects of this compound co-administrated with whole-body vibration training (WBVT), in the context of the knee and muscle function improvement, in elderly women with knee OA. In this double-blinded, placebo-controlled, randomized intervention study, the selected participants (26 females aged 65–85 years with knee OA) had WBVT and were allocated to receive either 16.7 mg of MA or a placebo daily for a period of 20 weeks. The outcomes of whole-body vibration training were compared with the respective placebo (WBVT/P) and MA (WBVT/MA) groups. Based on the results of this analysis on muscle function measurements, positive outcomes were reported in all groups, underlining the significance of a non-invasive (chemical or natural treatment) approach, while there was significant interaction (time × group) (*p* = 0.03) in the "isokinetic extension peak torque" domain for severe OA (Kellgren and Lawrence (K-L) grade ≥ 3) [128]. Moreover, in the setting of an open-label clinical trial, daily consumption of a MA-containing product (30 mg MA) for a period of 16 weeks improved the physical quality of life, level of bodily pain, and physical component, but not mental quality of life in a total of 29 elderly (mean age: 70.7 ± 10.1 years) participants [129].

#### 3.3.6. Pomegranate

Pomegranate extracts have been found to have strong anti-inflammatory, antioxidant, and even antitumor properties in vivo and in vitro, while there is also some evidence supporting this natural product in a clinical setting. Namely, data from a placebo-controlled trial showed that the intervention (500 mg of pomegranate peel, hydro-alcoholic extract) increased Knee injury and Osteoarthritis Outcome Score (KOOS) mean from 46.15 ± 16.82 to 57.57 ± 15.61 after 12 weeks (*p* < 0.001) as compared to the placebo group (baseline: 50.83 ± 18.83, after 12 weeks: 56.10 ± 18.07) (*p* < 0.001), while the VAS score remarkably declined in both arms compared with the respective baselines (*p* < 0.005) [130]. Similar results were observed in a smaller scale study involving 38 patients with knee OA, who were allocated to receive pomegranate juice and placebo for 6 weeks. This 2016 study showed that the WOMAC index total score (*p* = 0.01), stiffness score (*p* = 0.00), and physical function score (*p* = 0.01) were scientifically decreased in the intervention group, as well as the means MMP-13 levels (*p* = 0.02), while glutathione peroxidase was increased (*p* = 0.02) [131].

#### 3.3.7. Spearmint (*Mentha spicata*) 

Mints (*Mentha* sp.) have been among the most widely used aromatic plants for food flavoring, tea preparation with anti-inflammatory, anti-carcinogenic, antioxidant, and anti-peroxiding properties [132]. The potential of a high-rosmarinic acid (rosA) spearmint tea was studied in the setting of a randomized, double-blind study on KOA patients. Data from 46 participants (mean age = 60.7; BMI = 32.9 kg/m^2^) showed that pain score considerably decreased from week 0 to 16 for the high-rosA group instead of the control group, as well as scores for physical disability and stiffness for both groups. Additionally, increased quality of life (QoL) score on the bodily pain index in the SF-36 was observed at week 16 within the high-rosA group only, as well as a non-significant improvement in the six-minute walking test (6MWT) [133]. 

#### 3.3.8. Turmeric (*Curcuma domestica*) (*Curcuma longa*) 

Turmeric (curcumin) is generally considered the most biochemically effective compound derived from the rhizomes of *Curcuma* spices [134]. A 2014 study, involving 367 primary KOA patients randomly assigned to an active comparator or *C. domestica* extracts for 4 weeks, showed overall improvement in WOMAC scores of the treatment group versus the baseline, while events of abdominal pain/discomfort were more often in the ibuprofen (positive control) group (*p* = 0.046), and most of the participants declared improvement of their overall status [135]. In a recent study, KOA participants (n = 40) were divided to receive encapsulated curcuminoids (1500 mg/day*3; n = 19) and placebo capsules (n = 21) [136]. This 6-week intervention with curcuminoids showed an increase in glutathione levels (*p* = 0.064) and SOD activity (*p* < 0.001) in serum [136]. A summary of the overall clinical findings of this review is presented in Table 3. 

## 4. Discussion

In this study, we evaluated the literature over the last decade regarding the potential anti-inflammatory role of natural products against osteoarthritis. We evaluated all the references regarding natural products and pure natural compounds (using in vitro, in vivo, ex vivo data, and animal models) and clinical trials on humans. 

This review overall presented the findings of almost 90 published papers, most of which documented the anti-inflammatory properties of natural products and natural compounds in vitro. A total of 17 natural products and 14 isolated compounds are found to have reported anti-inflammatory and/or chondroprotective activity, along with properties of pain reduction and physical function improvement. 

More specifically, in 59 studies, 12 natural products and 13 pure natural compounds were evaluated in vitro, mostly by employing assays on RAW 264.7 or chondrocytes, while, in three of them, the pure compounds were studied via animal models. One study presented ex vivo results.

At the molecular level, the inhibition of several inflammatory mediators (MMPs, PGE_2_, IL-6, NO, iNOS, COX-1-2, LOX, and TNF-α, to name a few) and NF-κB and MAPKs pathway activation are commonly found among the results of in vivo findings; however, others, such as NOP, SOD, MDA or TIMP-1, COMP, MPO, OPRL1, and ADAMTS-4-5, are not frequently assessed. This might be a key indicator as to why much promising natural products or compounds based on in vitro and in vivo findings tend to fail in clinical trials. On that note, curcumin, resveratrol, maslinic acid, and some anthocyanins are bright examples of the opposite, with occasionally limited yet crucial clinical evidence of their effects against osteoarthritis. 

An interesting remark regarding the action of all these different compounds, at a molecular level, is the observation of a not so diverse impact on such a vast variety of molecules. On that note, C-terminal telopeptide of collagen type II has been one of the most frequently assessed biomarker of collagen metabolism, and cartilage oligomeric matrix proteins (COMP and its deamidated form D-COMP) and matrix metalloproteinases (MMP-1, MMP-3, MMP-9, MMP-13, and TIMPs) have been the most well-known biomarkers related to other non-collagenous proteins and regarding biomarkers related to other processes; the inflammatory biomarkers IL-1β, IL-6, and COX-2 have been mostly evaluated. However, also significant biomarkers in OA, such as type II collagen pro-peptides (PIINP, PIIANP, PIIBNP, PIICP, CPII), pyridinoline, Glc-Gal-PYD, collagen type II-specific neoepitope (C2M), N-terminal telopeptide of collagen type I (NTX-I), core protein fragments (aggrecan neoepitopes, ARGS(amino acids alanine (Ala; A), arginine (Arg; R), glycine (Gly; G) and serine (Ser; S); N-terminal sequence created by aggrecanase cleavage), and FFGV fragments), fibulin (peptides of fibulin 3, Fib3-1, Fib3-2), follistatin-like protein 1 (FSTL-1), soluble receptor for advanced glycation end-products (sRAGE), adipokines (adiponectin, leptin, visfatin), soluble receptor for leptin (sOB-Rb), cellular interactions in bone (periostin), and Wnt inhibitors (DKKs and SOST) are rarely evaluated. Additionally, as previously reported, only the NF-kappa B, the Phosphatidylinositol 3-kinase/ Protein kinase B (PI3K/Akt), and the MAPK signaling pathways have mostly been considered for analysis as the most well-known pathways in the development or progression of OA. However, research has shown that there might be a lot more to explore than these, namely, the renin secretion, apoptosis, the TNF signaling pathway, calcium reabsorption, the cGMP-Protein kinase G (PKG) signaling pathway, the adipocytokine signaling pathway, the estrogen signaling pathway, glycerophospholipid metabolism, the thyroid hormone signaling pathway, and the cAMP signaling pathway. The understanding of this elaborate network of pathways and implicated biomarkers in the progression of osteoarthritis can unravel the targets of the different natural products or compounds with a hope to highlight their true therapeutic potential. 

In a total of 12 clinical trials, three natural compounds and eight natural products were evaluated, mostly against placebo and, in some cases, against active comparators (i.e., ibuprofen) for intervention periods ranging from 4 to 20 weeks. It is remarkable that in all cases, there was the oral treatment of either the natural products or the pure natural compounds. Natural occurring products present significant advantages in oral treatment in terms of safety and bioavailability, which has to be further investigated for their potential use in prevention and treatment against osteoarthritis.

Considering that inflammation and pain are the major problems for OA patients, particularly for those with knee osteoarthritis, which can dramatically affect the quality of life, several investigations are focusing on this area, hoping to provide solutions. The utilization of complementary and alternative medicine in the treatment of a variety of medical conditions is an increasingly observed phenomenon. The clinical use of nutritional modulators as a treatment of pain has already been widely studied in cases of myalgias, rheumatoid arthritis, menstrual pain, and osteoarthritis. Although some nutritional modulators are endorsed via scientific research for their ability to contribute or treat in a variety of pain and inflammation states, others (including turmeric, devil's claw, methylsulfonylmethane (MSM), Boswellia, white willow bark, and green tea) have contradictory or minimal evidence to support their use for inflammation relief. Despite the medical evidence or lack thereof, passionate consumers continue to utilize these modulators as supplements or alternatives to conventional pharmacotherapy [139].

It is interesting to be mentioned that most of the natural products, as well as the pure natural compounds, have already been further evaluated for other biological activity, providing a multi-target profile, which has to be considered for further utilization of natural products.

## 5. Conclusions

Our work has highlighted the need for consistency in the findings, which can only be achieved through a deeper understanding of the mechanisms involved in the development of OA and those that nutritional mediators can affect, by also considering dose and time factors. Given the significant content of dietary polyphenols and other natural products in the typical human diet and the potential of dietary supplements, more well-designed human clinical trials are needed to evaluate the effects of natural products and compounds thereof on OA in terms of functional, structural, and biochemical outcomes. 

## Figures and Tables

**Table 1:** Inclusion/exclusion criteria of the studied papers.

Criterion	Inclusion	Exclusion
Study Design	In vitro studies, *Ex vivo* studies, Animal models, Clinical trials (Randomized control trials, Observational, Prospective, Single-arm, Non-randomized) (Note 2), Meta-analysis of RCTs	Reviews (Note 1) Case studies, Commentaries, Editorials letters, News, Notes, Guidelines Conference abstracts
Intervention	Whole food (i.e., herbs, fruits, vegetables) Extracts (i.e., plants, herbs) Essential oils Pure compounds (i.e., resveratrol, quercetin)	Non-natural products Pharmaceutical agents (Note 4) Processed food Exercise (alone) Operational or other medical intervention as primary treatment (Note 3) Acupuncture treatment Chiropractic manipulation or other alternative therapies (relaxation techniques and hydrotherapy)
Comparator	Placebo Non-natural products Pharmaceutical agents (Note 4) Exercise, Acupuncture, Chiropractic manipulation (alone or in combination with the intervention)	Operational or other medical intervention as primary treatment (Note 3) Assistive devices: scooters, canes, walkers, splints, shoe orthotics, etc. Any other comparator that is not mentioned in the inclusion list or cannot be otherwise justified for inclusion
Outcomes	Improvement of inflammation, managing symptoms (Note 5)	Other outcomes not reported or cannot be derived
Language	English	Non-English full text available
Time frame	2008–2019	Earlier than January 2008

**Table 2:** Natural compounds with effects on the OA setting (Summary).

Compound	Effect/-s	Cell-line	Animal Model	Ref.
Curcumin Curcumin + Boswellia serrata	Down-regulation of OPRL1, ADAMTS5 Suppresses NOP production	Human T98G neuroglia cells		[97]
Curcumin + Ginger	Decreases COMP, HA, MPO, MDA, and IL-1beta levels SOD elevation Reduction of MDA levels		MIA-induced OA in rats	[98]
Thymoquinone	Inhibition of COX-2, iNOS, NO, and PGE_2_ production, NF-κB activation, and IκBα degradation Suppresses MMP-1, MMP-3, and MMP-13 production and MAPK activation	Human OA chondrocytes		[104]
Geniposide	Attenuates the expression of IL-1, TNF-α, and NO production, MMP-13 in chondrocytes	Rabbit OA chondrocytes		[99]
	Suppresses the expression of IL-1, TNF-α, NO, and MMP-13 in the synovial fluid	Rat OA chondrocytes		[100]
	Promotes increased production of collagen II Decreases the expression of proapoptotic molecules—Bax, Cyto-c, and C-caspase 3 Increases Bcl-2 expression			
Oleuropein	Inhibits the production of NO and PGE_2_ Reduces the expression of COX-2, iNOS, MMP-1, MMP-13, and ADAMTS-5 Suppresses the degradation of aggrecan and collagen-II Suppresses NF-κB and MAPK activation	Human OA chondrocytes		[101]
Anthocyanins cyanidin-3-O-glucoside peonidin-3-O-glucoside protocatechuic Acid	Attenuate the inhibition of porcine cartilage degradation	Human OA chondrocytes	Porcine	[105]
	Inhibit the production of MMPs Inhibit IκBα degradation Reduce phosphorylation of p65 Suppress the ERK/MAPK pathway Enhance the phosphorylation of JNK			
Malvidin	Pain-relieving effects Decreases the expression level of apoptotic marker SA-beta-gal in chondrocytes		MIA-induced OA in rats	[106]
	Inhibits expressions of IL-1B, IL-6, TNF-α, and MMPs Suppresses NF-κB pathway activation			
Chrysin	Inhibits the production of NO and PGE_2_ Reduces the expression of COX-2, iNOS, MMP-1, MMP-13, and ADAMTS-5 Suppresses the degradation of aggrecan and collagen-II Suppresses NF-κB and MAPK activation	Human OA chondrocytes		[107]
Astragalin	Inhibits the production of NO and PGE_2_ Reduces the expression of COX-2, iNOS, MMP-1, MMP-13, and ADAMTS-5 Suppresses the degradation of aggrecan and collagen-II Suppresses NF-κB and MAPK activation Activates PPAR-γ	Human OA chondrocytes		[108]
Isorhamnetin	Inhibits the expression of stromelysin-1 and collagenase 3 Suppresses the production of NO and PGE_2_, iNOS, and prostaglandin G/H synthase 2 Reduces the expression of NF-κB and transcription factor p65 Attenuates the degradation of NF-κB inhibitor α	Human chondrocytes		[109]
Resveratrol	Suppresses IL-1β-induced proteasome function and the degradation of IκBα without affecting IκBα kinase activation, IκBα-phosphorylation, or IκBα-ubiquitination Inhibits IL-1β-induced apoptosis, caspase-3 activation, and PARP cleavage	Human articular chondrocytes		[110,111,112]
	Reduces cell viability of IL-1β-stimulated chondrocytes, expression levels of MMP-13, IL-6, TNF-α, p-IRAK4, TLR4-TRAF6, and pain	Human OA chondrocytes		[113]
	Alleviates OA pathology by decreasing the levels of systematic inflammation and/or inhibiting TLR4 signaling pathway in cartilage		OA rats (on a high-fat diet)	[114]
	An inhibitory effect on the pro-inflammatory marker secretionDecrease of IL-6 secretion -> NFκB inhibition in chondrocytes -> limits STAT3 activation in the macrophages Interruption of the inflammatory amplification loop	Chondrocytes and macrophages co-culture		[115]
	Suppresses AGEs-induced expression of iNOS and COX-2 and production of NO and PGE_2_	Porcine chondrocytes and cartilage explants		[116]
	Decreases AGEs-stimulated expression and activity of MMP-13 and prevents AGEs-mediated destruction of collagen II			
Rosmarinic acid	Increases type II collagen, sulfated-proteoglycan, COX-2, and PGE_2_ production Suppresses expression of MMP-13	Rabbit OA chondrocytes		[117]
	Inhibits the production of IL-6, gene and protein expression of ADAMTS-4 and ADAMTS-5 Averts the inhibition of ACAN and COL2 gene expression	Rat OA chondrocytes		[118]
Schisantherin A	Inhibits the production of NO, PGE_2_, and TNF-α Suppresses the expression of MMP-1, MMP-3, and MMP-13 Inhibits NF-κB and MAPKs activation	Human OA chondrocytes		[119]
Matrine	Inhibits IL-1beta-induced apoptosis Decreases MMP-3 and MMP-13 expression Suppresses the phosphorylation of p-38 and IκB-α degradation Suppresses ERK/MAPK pathway Enhances the phosphorylation of JNK Increases the production of TIMP-1	Human OA chondrocytes		[121]
Diosgenin	Inhibits the production of NO and PGE_2_ Reduces the expression of MMP-3 and MMP-13 Decreases iNOS and COX-2 expression Suppresses degradation of IκB-α	Human OA chondrocytes		[120]

ACAN: Aggrecan; ADAMTS-5: ADAM metallopeptidase with thrombospondin type 5 motif; ADAMTS-4: ADAM metallopeptidase with thrombospondin type 4 motif; AGEs: Advanced glycation end products; Bcl-2: B-cell lymphoma 2; COMP: Cartilage oligomeric matrix protein; COX: Cyclooxygenases; CTX-II: C-telopeptide of type II collagen; ECM: Extracellular matrix; ERK: Extracellular signal-regulated kinases; GAG: Glycosaminoglycan; HA: Hyaluronic acid; IL: Interleukin; IMG: Isomucronulatol 7-O-β-d-glucoside; iNOS: Inducible nitric oxide synthase; IκBα: inhibitor of kappa B; JNK: c-Jun N-terminal kinase; LOX: Lipoxygenase; LPS: Lipopolysaccharide; LTB4: Leukotriene B4; MAPK: Mitogen-activated protein kinase; MDA: Malondialdehyde; MIA: Monosodium iodoacetate; MKK3: Mitogen-activated protein kinase kinase-3; MMP: Matrix metalloproteinases; MPO: Myeloperoxidase; NF-κB: Nuclear factor-kappa B; NO: Nitric oxide; NOP: Nociceptin opioid peptide; OPRL1: Opioid-related nociceptin receptor 1; PARP: Poly (ADP-ribose) polymerase; PGE2: Prostaglandin E2; PIINP: Procollagen II N-terminal pro-peptide; p-IRAK4: Phospho-interleukin-1 receptor-associated kinase 4; PPAR-γ: Peroxisome proliferator-activated receptor gamma; ROS: Reactive oxygen species; RUNX-2: Runt-related transcription factor 2; SA-beta-gal: Senescence-associated beta-galactosidase; SOD: Superoxide dismutase; STAT3: Signal transducer and activator of transcription 3; TLR4: Toll-like receptor-4; TNF-α: Tumor necrosis factor alpha; TRAF6: Tumor necrosis factor receptor-associated factor 6.

**Table 3:** Clinical trials investigating the effects of natural products and compounds in OA (Summary).

Natural product/Form	Trial Type/Duration/ Trial ID	Population	Intervention Groups/Participants (n)	Observations	Adverse Events (AEs)	Ref.
Acacia catechu (heartwood) and Morus alba (root bark) - Standardized blend (UP1306)	Prospective, randomized, double-blind, comparator, and placebo-controlled parallel-group 12 weeks ISRCTN15418623	- 135 participants - 35–75 years old - BMI < 35 kg/m^2^ - Knee pain for at least 15 of the 30 days prior study - Symptoms of knee pain for at least 6 months before the study - Kellgren–Lawrence grade of I, II, or III (screening X-ray)	- UP1306 (100 mg) (n = 45) - Glucosamine (375 mg per capsule) and Chondroitin (300 mg) combination (n = 45) - Placebo (n = 45)* Applicable for each treatment group: Dose was four capsules per day	- WOMAC-pain decreased by 51% (SD = 30) for UP1306, by 45% (SD = 41) for glucosamine/chondroitin, and by 46% (SD = 40) for placebo (*p* = 0.753 between groups) - WOMAC-pain decreased from baseline to day 56 for UP1306 vs. glucosamine/chondroitin (*p* = 0.048 between groups) - WOMAC-stiffness decreased by 45% (SD = 37) for UP1306, by 38% (SD = 45) for glucosamine/chondroitin, and by 56% (SD = 37) for placebo (*p* = 0.115 between groups) - VAS-discomfort ratings were not statistically significant between the three products (*p* = 0.935). - VAS-discomfort ratings decreased from baseline to all time-points for all treatment groups (within-group differences) - Rescue medication overall use was 18.8% (SD = 17.9) for UP1306, 19.0% (SD = 21.0) for glucosamine/chondroitin, and 21.2% (SD = 17.1) for placebo - Increased extension scores from baseline to day 28 for glucosamine/chondroitin vs. UP1306 (*p* = 0.018) and placebo vs. UP1306 (*p* = 0.042) - Distance walked (6MWT) increased within-groups from baseline to all time-points. No significant differences between groups (*p* = 0.183) - TNFα levels: No significant differences from baseline to 12 weeks between groups (*p* = 0.786) - Urinary CTX-II levels: Significant difference for UP1306 and placebo after 12-weeks (*p* = 0.029)	- No serious adverse events - 43 adverse events were observed among 30 of the 133 subjects (safety population) - 15 AEs UP1306 group - 10 AEs glucosamine/chondroitin - 18 AEs placebo - Gastrointestinal disorders and infections and infestations were most frequently reported.	[124]
Litsea japonica Extract	Randomized, double-blind, placebo-controlled 12 weeks KCT0001029	- 87 participants - 50–70 years old - Painful knee OA	- Placebo group - Low-dose group - 100 mg/d - High-dose group - 200 mg/d	- VAS scores- pain reduction (*p* = 0.0001) - WOMAC - total (*p* = 0.0102) - WOMAC - pain (*p* = 0.0293) - WOMAC - stiffness (*p* = 0.002) - WOMAC - function (*p* = 0.0152) - Blood MMP-9 levels (*p* = 0.026)	Not Reported	[58]
Pomegranate - *Punica granatum L.* (sweet Malas verity) (Peels) - Ethanol/Water extract was encapsulated with rice flour (500 mg of PPE with 50 mg flour)	Randomized, double-blind, placebo-controlled 8 weeks IRCT201405183664N11	- 66 female adult participants - Moderate knee OA - 38–60 years old - BMI: 30–35 kg/m^2^	- Placebo (550 mg rice flour): 2 capsules/d (n = 33) - Pomegranate (500 mg): 1 capsule/d (n = 33)	- KOOS scores increased compared with baseline in intervention group (Mean ± SD before treatment = 46.15 ± 16.82 to 57.57 ± 15.61 after treatment, *p* < 0.05) - VAS score decreased compared with baseline in the intervention group (*p* < 0.05)	Not Observed	[130]
Pomegranate Juice	Randomized, parallel-arm 6 weeks	- 39 adult participants - 30–80 years old - Knee OA	- Control (n = 19) - Pomegranate juice: 200 mL/d (n = 20)	- Decreased WOMAC total score (from 39.63 ± 18.89 to 32.36 ±16.62, *p* = 0.01) after treatment - Decreased WOMAC - stiffness score (from 2.58 ± 2.29 to 1.47 ± 1.80, *p* = 0.00) after treatment - Decreased WOMAC - function score (from 27.74 ± 10.56 to 22.53 ± 10.91, *p* = 0.01) after treatment - Decreased WOMAC - pain score (from 7.95 ± 4.99 to 7.32 ± 4.95, *p* = 0.49) after treatment - MMP-1 increases in serum were not significant in the treatment group compared to baseline - Means of MMP-13 serum levels decreased (from 157.81 ± 66.22 to 127.99 ± 34.04, *p* = 0.13) after treatment - Glutathione peroxidase serum levels increased in the treatment group vs. control group (*p* = 0.02)	Not Observed	[131]
Spearmint - High-rosmarinic acid (rosA) spearmint tea	Randomized, double-blind, parallel-arm 16 weeks NCT01380015	- 62 adult participants - Nonsmokers - Knee OA - WOMAC pain score >125 (to be included in the study)	- Spearmint tea high-rosmarinic acid (130–150 mg per cup) (2 cups/d) (n = 28) - Control tea low - rosmarinic acid (13 mg per cup) (2 cups/d) (n = 28)	- WOMAC - pain decreased from baseline in the treatment group (*p* = 0.002) - WOMAC - stiffness decreased from baseline in the treatment group (*p* = 0.004) - WOMAC - physical disability decreased from baseline in the treatment group (*p* = 0.02) - WOMAC scores on all scales were not significantly different between groups - SF-36 QoL score (bodily pain) increased from 46.6 ± 7.6 (baseline) to 58.1 ± 22.1 (week 16) in the treatment group (*p* < 0.05) - Six-minute walk test change from baseline was +22.3 m for the treatment group and +0.1 m for the control group with no significant differences within or between groups	- Overall, no serious adverse events reported - AEs in treatment group: constipation (n = 3), headache (n = 2), loose bowel movements (n = 1) - AEs in control group: staining of dentures (n = 1), dry mouth (n = 1), itchy skin (n = 1)	[133]
*Curcuma domestica* - Extract (encapsulated)	Randomized, double-blind, controlled (multicenter) 4 weeks NCT00792818	- 367 adult participants - Older than 50 years of age - Knee OA - NRS of knee pain of ≥5 out of 10	- Control: Ibuprofen (1200 mg/d) (n = 182) - C. domestica extracts (1500 mg/d) (250 mg of curcuminoids) (n = 185)	- All WOMAC scores improved from baseline (*p* < 0.001) in both groups - WOMAC - total, noninferiority test, treatment vs. control (*p* = 0.010) - WOMAC - pain, noninferiority test, treatment vs. control (*p* = 0.018) - WOMAC - function, noninferiority test, treatment vs. control (*p* = 0.010) - WOMAC - stiffness, noninferiority test, treatment vs. control trended toward significance (*p* = 0.060) - 6MWT: No difference between groups (Mean difference (95% CI) = 7.18 (−7.01, 21.38)), *p* = 0.320)	- AEs in treatment group = 1 - AEs in control group = 6	[135]
*Curcuma longa* and *Boswellia serrata* - Extracts formulation (encapsulated)	Randomized, single-blind, controlled 12 weeks	- 28 adult participants - 18–65 years old - Knee OA (moderate)	- Control: Celecoxib (100 mg/d) *2 (n = 14) - Curcuma longa and Boswellia serrata (500 mg/d) *2 (n = 14)	- OA joint pain: Patients in category moderate/severe in treatment group improved from 85.71% to 21.43% after treatment - OA joint pain: Patients in category moderate/severe in the control group decreased from 78.57% to 50% after treatment - Walking distance >1000 m was reported for 92.86% of patients in the treatment group and 85.71% in the control group	Not observed	[137]
Maslinic acid (Scheme 4) - Extract formulation (approx. 10.7% maslinic acid)	Randomized, double-blinded, placebo-controlled 20 weeks	- 26 female adult participants - 65–85 years old - Pain in one or both knees - BMI mean = 23.7 kg/m^2^	- Placebo (3 capsules/d) (n = 15) - Maslinic acid (3 capsules/d) (n = 11)	- Muscle function: isokinetic extension peak torque - time * group interaction (*p* = 0.03), effect sizes were higher in the treatment group for all domains of muscle function - Knee function: time was a significant factor in the domain of "pain on ascending or descending stairs” (*p* < 0.01) Kellgren and Lawrence (K-L) grade had a main effect in “pain on walking” (*p* = 0.04) and “range of motion” (*p* < 0.01) - Performance of WBVT program: Participants in the treatment group attended 422 of 440 training hours Participants in the control group attended 581 of 600 training hours	Not reported	[128]
Maslinic acid (Scheme 4) - Olive fruit extract formulation (jelly, approx. 10.4% maslinic acid)	Open-label (single arm) 16 weeks	- 35 adult participants - Chronic pain of the knee	-Maslinic acid 30 mg/d	- VAS - pain: decreased from baseline to 4 weeks after treatment (29.1 ± 26.8 to 24.5 ± 29.4, *p* = 0.06) - VAS - pain: decreased from baseline to 16 weeks after treatment (29.1 ± 26.8 to 22.5 ± 22.3, *p* = 0.08) - Japanese knee osteoarthritis measure (JKOM): decreased from baseline to 8 weeks after treatment (23.1 ± 18.5 to 19.9 ± 19.9, *p* = 0.04) - Short form-8 health survey (SF-8): Quality of life (QoL) was improved after treatment. All domains of the SF-8 survey had significant improvement compared to baseline at one or more time points (4, 8, or 16 weeks after treatment)	Not observed	[129]
Resveratrol (Scheme 4) - Synthetic trans-resveratrol (encapsulated)	Randomized, double-blind, placebo-controlled 14 weeks ACTRN12615000291583	- 80 female adult participants - 45–85 years old - Postmenopausal - Nonsmokers - Age-related osteoarthritis - BMI = 26.7 ± 0.6 kg/m^2^	- Resveratrol (75 mg*2/d) (n = 37) - Placebo * 2/d (n = 35)	- CVR to hypercapnia improved in both groups (Treatment group results at 14 weeks: 58.1 ± 2.4%, *p* = 0.011. Placebo group results at 14 weeks: 52.4 ± 2.3%, *p* = 0.011) - VAS - pain: overall reduced by 10% in the treatment group vs. placebo	Not Observed	[138]
Coriander (*Coriandrum sativum L*.) leaf powder	Open-label 8 weeks	- 20 adults (OA diagnosis) - 20 adults (Healthy controls) - 40–60 years old	- Coriander leaf powder (5 g/d) (n = 40)	- ALP: Decreased by 5% (*p* < 0.01) after treatment (from 107 ± 0.37 to 102 ± 2.19, Mean ± SEM, intervention group) - ALP: Increased by 20% in the control group-Calcium: Increased by 6% (*p* < 0.05) after treatment (from 5.79 ± 0.11 to 6.15 ± 0.03, Mean ± SEM, intervention group)-Calcium: Decreased by 15% in the control group - ESR: Decreased by 64% (*p* < 0.01) after treatment (from 36.6 ± 2.3 to 13.3 ± 3.8, Mean ± SEM, intervention group) - ESR: Decreased by 18% in the control group	Not Reported	[126]
Guilu erxian jiao	Randomized, Case-control 12 weeks	- 21 male adults (treated group) - 21 male adults (untreated group) - Older than 65 years old - Knee OA	- Guilu erxian jiao (6 g/d) (n = 21)	- VAS – pain improvement (*p* < 0.01) after treatment - Reduced Lequesne index scores: pain/discomfort (*p* < 0.01), maximum distance walked (*p* < 0.01), activities of daily living (*p* < 0.05)	Not Observed	[45]

AEs: Adverse Events; ALP: Alkaline phosphatase; BMI: Body Mass Index; CTX-II: C-telopeptide of type II collagen; CVR: cerebrovascular responsiveness; ESR: Erythrocyte sedimentation rate; JKOM: Japanese knee osteoarthritis measure; K-L: Kellgren and Lawrence scale; KOA: Knee Osteoarthritis; KOOS: Knee injury and Osteoarthritis Outcome Score; MMP: Metalloproteinase; OA: Osteoarthritis; PPE: Pomegranate peel extract; QoL: Quality of Life; SF-8: Short form-8 health survey; TNF-a: Tumor necrosis factor-alpha; VAS: Visual analog scale; WBVT: Whole Body Vibration Training; WOMAC: Western Ontario McMaster universities osteoarthritis index; 6MWT: Six minutes walking test. SD: Standard Deviation; SEM: Standard Error of Means.

## Abbreviations

ACLT: Anterior cruciate ligament transection; ADAMTS: ADAM metallopeptidase with thrombospondin type 1; AGEs: Advanced glycation end products; Bcl-2: B-cell lymphoma 2; COMP: Cartilage oligomeric matrix protein; COX: Cyclooxygenases; CTX-II: C-telopeptide of type II collagen; ECM: Extracellular matrix; ERK: Extracellular signal-regulated kinases; GAG: Glycosaminoglycan; HA: Hyaluronic acid; IL: Interleukin; IMG: Isomucronulatol 7-O-β-d-glucoside; iNOS: Inducible nitric oxide synthase; IκBα: inhibitor of kappa B; JNK: c-Jun N-terminal kinase; LOX: Lipoxygenase; LPS: Lipopolysaccharide; LTB4: Leukotriene B4; MAPK: Mitogen-activated protein kinase; MDA: Malondialdehyde; MIA: Monosodium iodoacetate; MKK3: Mitogen-activated protein kinase kinase-3; MMP: Matrix metalloproteinases; MPO: Myeloperoxidase; NF-κB: Nuclear factor-kappa B; NO: Nitric oxide; NOP: Nociceptin opioid peptide; OPRL1: Opioid-related nociceptin receptor 1; PARP: Poly (ADP-ribose) polymerase; PGE_2_: Prostaglandin E_2_; PIINP: Procollagen II N-terminal pro-peptide; p-IRAK4: Phospho-interleukin-1 receptor-associated kinase 4; PPAR-γ: Peroxisome proliferator-activated receptor gamma; ROS: Reactive oxygen species; RUNX-2: Runt-related transcription factor 2; SA-beta-gal: Senescence-associated beta-galactosidase; SOD: Superoxide dismutase; STAT_3_: Signal transducer and activator of transcription 3; TLR4: Toll-like receptor-4; TNF-α: Tumor necrosis factor alpha; TRAF6: Tumor necrosis factor receptor (TNFR)-associated factor 6; VAS: Visual analog scale; WOMAC: Western Ontario McMaster universities osteoarthritis index
